# The influence on quality of life of intermittent scheduling in first- and second-line chemotherapy of patients with HER2-negative advanced breast cancer

**DOI:** 10.1007/s10549-019-05495-3

**Published:** 2019-11-28

**Authors:** Anouk K. M. Claessens, Reinier Timman, Jan J. Busschbach, Jeanette M. Bouma, Jeany M. Rademaker-Lakhai, Frans L. G. Erdkamp, Vivianne C. G. Tjan-Heijnen, Monique E. M. M. Bos, the Dutch Breast Cancer Research Group (BOOG)

**Affiliations:** 1 Department of Medical Oncology, Zuyderland Medical Centre, Dr. H. van der Hoffplein 1, 6162 BG Geleen, The Netherlands; 2 Department of Psychiatry, Section of Medical Psychology & Psychotherapy, Erasmus Medical Centre, Doctor Molewaterplein 40, 3015 GD Rotterdam, The Netherlands; 3 Department of Trial Registration, Comprehensive Cancer Centre the Netherlands, Vasteland 78, 3011 BN Rotterdam, The Netherlands; 4 Dutch Breast Cancer Research Group, BOOG Study Center, IJsbaanpad 9, 1076 CV Amsterdam, The Netherlands; 5 Department of Medical Oncology, GROW – School for Oncology and Developmental Biology, Maastricht University Medical Centre, P. Debyelaan 25, 6229 HX Maastricht, The Netherlands; 6 Department of Medical Oncology, Medical Oncologist, Erasmus Medical Centre, Doctor Molewaterplein 40, 3015 GD Rotterdam, The Netherlands

**Keywords:** Quality of life, Advanced breast cancer, Chemotherapy, Scheduling

## Abstract

**Background:**

The Stop&Go study randomized patients with advanced breast cancer to intermittent (two times four) or continuous (eight subsequent cycles) first- and second-line chemotherapy.

**Methods:**

QoL was measured with RAND-36 questionnaires every 12 weeks. The primary objective was to estimate differences in changes from baseline between intermittent and continuous treatment. An effect size of 0.5 SD (5 points) was considered clinically meaningful.

**Results:**

A total of 398 patients were included with a median follow-up of 11.4 months (IQR 5.6–22.2). Mean physical QoL baseline scores were 38.0 resp. 38.2, and mental scores 45.0 resp. 42.4 for intermittent and continuous treatment. Physical QoL declined linearly in the intermittent arm causing a clinically meaningful difference of 5.40 points at 24 months (*p* < 0.001), while scores in the continuous arm stabilized after a small decline of ± 3.4 points at 12 months. Conversely, mental QoL was fairly stable and even improved with 1.58 (*p* = 0.005) and 2.48 points (*p* < 0.001) at 12 months for intermittent and continuous treatment, respectively. When comparing arms for both components in changes from baseline, the maximum differences were 2.46 (*p* = 0.101) and 1.95 points (*p* = 0.182) for physical and mental scores, both measured at 30 months and in favor of continuous treatment.

**Conclusion:**

Intermittent first- and second-line chemotherapy in patients with HER2-negative advanced breast cancer showed a trend for worse impact on QoL compared to continuous chemotherapy, with neither significant nor meaningful differences in course. We recommend prescribing chemotherapy continuously until progressive disease or unacceptable toxicity.

*Trial registration* EudraCT 2010-021519-18; BOOG 2010-02

**Electronic supplementary material:**

The online version of this article (10.1007/s10549-019-05495-3) contains supplementary material, which is available to authorized users.

## Introduction

Breast cancer is the most common cancer among women worldwide [3]. Approximately 20–30% of all breast cancer patients will eventually develop metastases [4]. While the 5-year survival rate for women with breast cancer of all stages is estimated at 82%, survival rates for advanced breast cancer are relatively poor [5, 6]. Patients with advanced breast cancer (defined here as incurable locally advanced or metastatic breast cancer) experience a significant burden of disease [7]. The treatment goals for these patients are therefore to prolong survival and preserve Quality of life (QoL). The experienced QoL is determined by both tumor related complaints as well as the side effects of treatment. Different treatment strategies may improve survival and QoL, or keep either survival or QoL constant, while improving the other. It is also possible that QoL is improved, while survival is slightly reduced, or the other way around.

The Stop&Go study was a non-inferiority trial randomizing patients with advanced breast cancer to two sets of four cycles (intermittent) or eight consecutive cycles (continuous) of chemotherapy in first- and second-line treatment [8]. The hypothesis was that the intermittent treatment would improve QoL. Recently, we reported the first efficacy results from the Stop&Go trial, showing a lack of non-inferiority in progression-free survival (PFS) (medians 7.4 vs. 9.7 months, Hazard ratio [HR] 1.17; 95% CI 0.88–1.57), and shorter overall survival (OS) (medians 17.5 vs. 20.9 months, HR 1.38; 95% CI 1.00–1.91) for the experimental intermittent arm compared to the continuous arm in first-line treatment. [8] Here we report on the prospectively planned QoL analyses. If QoL is indeed better in the experimental arm, patients and physicians could make a trade-off between better QoL and better survival.

## Methods

### Study design and treatment

An extensive description of the study design has been published previously [8]. In brief, the Stop&Go study is a phase III trial assessing the impact of scheduling of first- and second-line chemotherapy in patients with HER2-negative advanced breast cancer. Participants were randomized to two sets of four cycles (intermittent) or eight consecutive cycles (continuous) of chemotherapy in first- and second-line treatment. First-line treatment consisted of paclitaxel plus bevacizumab, second-line treatment of capecitabine or non-pegylated liposomal doxorubicin.

### QoL assessments

QoL was measured by RAND-36 questionnaires [9], which were distributed by post at baseline and every 12 weeks during study treatment and follow-up until death or withdrawal due to any reason. Information about the social status of patient at randomization was collected retrospectively.

The questionnaire contains 36 multiple choice items divided over 8 subscales and is almost identical to the SF-36 [9, 10]. We first calculated the 8 subscales of the RAND-36 using the Dutch algorithm [11]. The weights of this algorithm to calculate T-scores (mean = 50, SD = 10) were obtained in several large Dutch samples (total *n* = 6822). As there is no Dutch algorithm for the calculation of physical and mental components of QoL out of the RAND-36, we then applied US weights used for the SF-36 to determine these component scores [12]. The physical and mental component scores are presented as T-scores with a mean of 50 and a standard deviation (SD) of 10 for the normal population, with higher scores representing better QoL. Individual missing items of the RAND-36 were handled using the algorithm presented by Ware [13].

### Outcomes

The evaluation of QoL comprised a secondary objective of the Stop &Go study. The primary, prospectively planned, objective was to describe the course of physical and mental QoL for both treatment arms, and to estimate differences in changes from baseline in physical and mental QoL between arms. Secondary objectives, added post hoc, were to assess the influence of age, Body Mass Index (BMI), living alone, Eastern Cooperative Oncology Group Performance Status (ECOG PS) at baseline, disease-free interval (DFI) between initial diagnosis and metastatic diagnosis, site of metastasis at baseline, receiving prior hormonal therapy for advanced disease, number and duration of (re-) hospitalizations, duration of first-line treatment, and receiving first- and/or second-line study treatment on QoL and compare these influences between treatment arms.

### Statistical analyses

QoL analyses included all patients within the Stop&Go study who responded to at least one questionnaire with sufficient valid data, according to the intention-to-treat principle. Baseline differences between study arms, dropouts and participants were analyzed with *χ*^2^ tests with continuity correction, *t* tests or Mann–Whitney *U* tests.

Differences in changes from baseline were estimated longitudinally with multilevel linear regression analyses. These analyses can handle data with unbalanced and incomplete time points, assuming missing at random, efficiently. For this multilevel approach, three levels were included; institute as upper level, the patients as middle level and their repeated measures as lower level. The deviance test was applied to determine the necessity of the institute level [14, 15]. For analyses of the treatment effect only, the fixed parts of the models included time, the logarithm of time, treatment group, and the interactions. For the secondary analyses of the covariate effects, separate models were postulated including these covariates with first- and second order interactions.

In order to prevent the few cases with very long follow-up measures to exert an unduly great influence on the models, we determined the 90th percentile of the maximum follow-up time per case. Analyses were performed within the period until this 90th percentile.

For the primary analyses a Bonferroni correction was applied and *p* values < 0.025 were considered significant. For the exploratory secondary analyses *p* values < 0.05 were maintained. Based on a systematic review about minimally important differences in health-related QoL studies, an effect size of 0.5 SD (in this case 5 points on the component scores) was considered clinically meaningful [16]. Additionally, we considered a change of 2 points on the component scores a small effect, 5 points a medium effect and 8 points a large effect. [17].

Post hoc power analyses estimated the minimum detectable effect size. We used the observed correlations between the measurements, an alpha of 0.025 and a power of 0.80. As the number of measurements declined rather fast, for each outcome two power analyses were performed: one based on two measurement points and one on four.

Statistical analyses were performed with IBM-SPSS version 24.

## Results

### Patient and treatment characteristics

**Fig. 1:**
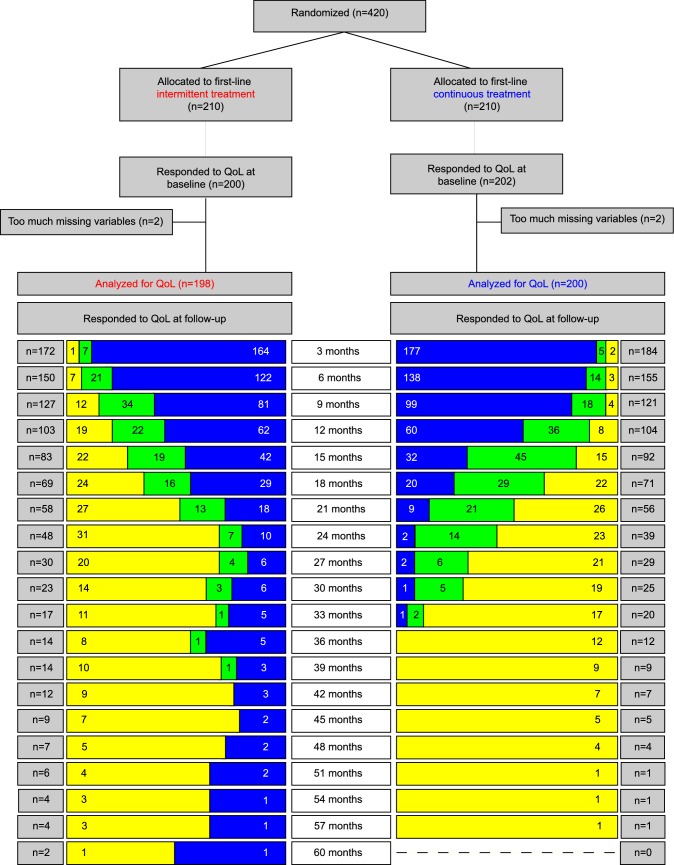
Flowchart of QoL response rates. The blue bars represent QoL measurements during first-line study treatment, the green bars represent QoL measurements during second-line study treatment, and the yellow bars represent QoL measurements during follow-up

At the time of data cut-off for the current analyses (May 1, 2018) a total of 420 patients were included and randomized in the Stop&Go study, of whom 402 (96%) responded to the QoL baseline questionnaire. Due to too much missing items or questionnaires, the population for analyses included 198 patients in the intermittent, and 200 patients in the continuous arm with at least one valid RAND-36 administration (total *n* = 398) (Fig. 1). There were no statistical significant differences in any of the patient- or treatment characteristics between the arms except for the DFI, which was longer for the intermittent treatment arm (Table 1). No significant difference was found in proportion of patients who continued on second-line treatment (Table 1, Fig. 1). Additionally, no significant differences were observed between the 22 non-responders compared to the 398 responders in treatment arm, early death, age and BMI (data not shown).

**Table 1:** (A) Baseline and (B) treatment characteristics in intermittent and continuous arms (ITT population, ***n***= 398)

(A) Baseline characteristics			
	Intermittent (*n* = 198)	Continuous (*n* = 200)	Total (*n* = 398)
Mean age in years (± SD)	58.2 (8.6)	59.0 (10.1)	58.6 (9.4)
Mean BMI (± SD)	26.1 (4.9)	26.3 (5.0)	26.2 (5.0)
Mean follow-up time in months (± SD)	13.3 (9.5)	13.4 (9.2)	13.3 (9.3)
Cohabited (%)	91/115 (79.1%)	101/129 (78.3%)	192/244 (78.7%)
ECOG PS^a^			
0–1	189/197 (95.9%)	186/200 (93.0%)	375/397 (94.5%)
2	8/197 (4.1%)	14/200 (7.0%)	22/397 (5.5%)
Site of metastatic disease			
Visceral	20 (10.1%)	18 (9.0%)	38 (9.5%)
Non-visceral	29 (14.6%)	25 (12.5%)	54 (13.6%)
Visceral and non-visceral	149 (75.3%)	157 (78.5%)	306 (76.9%)
Prior hormonal therapy for metastatic disease	94 (47.5%)	89 (44.5%)	183 (46.0%)

	Intermittent	Continuous	M–W Z	*p* value
Median DFI (months) between initial diagnosis and metastatic diagnosis [IQR]	55.0 [23.4–88.8]	39.1 [18.5–86.9]	− 1.592	0.111

(B) Treatment characteristics				
	Intermittent	Continuous	χ_(df)_^2^	*p* value
No. of patients with first-line treatment only (%)	71 (35.9%)	67 (33.5%)	0.151_(1)_	0.697
No. of patients with second-line treatment (%)	127 (64.1%)	133 (66.5%)		
			**M–W Z**	***p*value**
Median duration (months) until end of first-line treatment [IQR]	6.1 [4.4–12.5]	8.7 [5.3–11.4]	− 1.517	0.129
Median duration (months) until start second-line treatment [IQR]	7.8 [5.7–13.7]	10.5 [7.8–13.4]	− 2.017	*0.044*
Median total duration (months) of (re)hospitalizations [IQR]	3.0 [0.0–11.2]	4.3 [0.0–12.0]	− 0.773	0.439
Median number of (re)hospitalizations [IQR]	0.2 [0.0–1.4]	0.3 [0.00–1.4]	− 0.103	0.918

ITT, intention to treat; BMI, body mass index; ECOG PS, Eastern Cooperative Oncology Group Performance Status; ER, estrogen receptor; PgR, progesterone receptor; DFI, disease-free interval, IQR, inter quartile range; χ^2^, chi square; df, degrees of freedom, M–W Z, Mann**–**Whitney Z

Italic *p* values are considered statistically significant

^a^Missing ECOG PS: *n* = 1 vs. for intermittent arm

### Points of assessment

The maximum follow-up time was 68.2 months, with a median of 11.4 months (IQR 5.6–22.2). Median number of questionnaires per patient was 4.3 (IQR 2.1–7.5). Considering that 90% of the patients had a follow-up time within the range of 0.0 to 30.6 months, we limited the analyses to 30.6 months (Fig. 1, Supplementary Fig. S1). Drop-out of QoL assessments should take into account that active participants should administer a questionnaire every 12 weeks and may be overdue for half of that period. Thus at the end of the first year, participants should have responded after 34 weeks, or in case they deceased, should have responded at least 18 weeks before they died. At 12 months 297 women were still alive (75%), and 101 (25%) had deceased. Of the alive patients, 56 (19%) dropped out due to unknown reasons, while 21 of the deceased patients (21%) did not administer a questionnaire in their last 18 weeks of life, indicating reasons for drop-out other than death. At 24 months, 180 women were still alive (45%) and 218 had deceased (55%). A total of 76 alive participants (42%) dropped out, and 68 of the deceased (31%) dropped out at least 18 weeks prior to death. At the end of the study period at 30 months, 145 women were still alive (36%), and 253 had died (64%). Eighty-seven patients alive dropped out (60%) and 86 of the deceased patients (34%) dropped out prior to death.

### Changes in QoL over time

The deviance test pointed out that the institute level was superfluous (*χ*_(1)_^2^ = 2.22, *p* = 0.136). The estimates of the physical and mental component model for the main treatment effect without covariates at the various time points are presented in Table 2. Mean baseline scores were 38.0 and 38.2 for physical, and 45.0 and 42.4 for mental QoL with intermittent and continuous treatment, respectively. The model itself is presented in Supplementary Table S1.

**Table 2:** Estimated physical and mental components of QoL scores

	Intermittent				Continuous			
Months	0	12	24	30	0	12	24	30
**Physical component**	37.96	35.13	32.57	31.30	38.17	34.78	34.14	33.97
Difference with baseline	0.00	− 2.84	− 5.40	− 6.66	0.00	− 3.39	− 4.04	− 4.20
*p* value		0.001	< 0.001	< 0.001		< 0.001	< 0.001	< 0.001
Difference continuous–intermittent	0.00	0.55	− 1.36	− 2.46				
*p* value		0.542	0.265	0.101				
**Mental component**	44.95	46.53	44.90	43.89	42.43	44.91	43.99	43.33
Difference with baseline	0.00	1.58	− 0.05	− 1.06	0.00	2.48	1.55	0.89
*p* value		0.005	0.601	0.856		< 0.001	0.002	0.024
Difference continuous–intermittent	0.00	− 0.89	− 1.60	− 1.95				
*p* value		− 0.083	− 0.150	− 0.182				

**Fig. 2:**
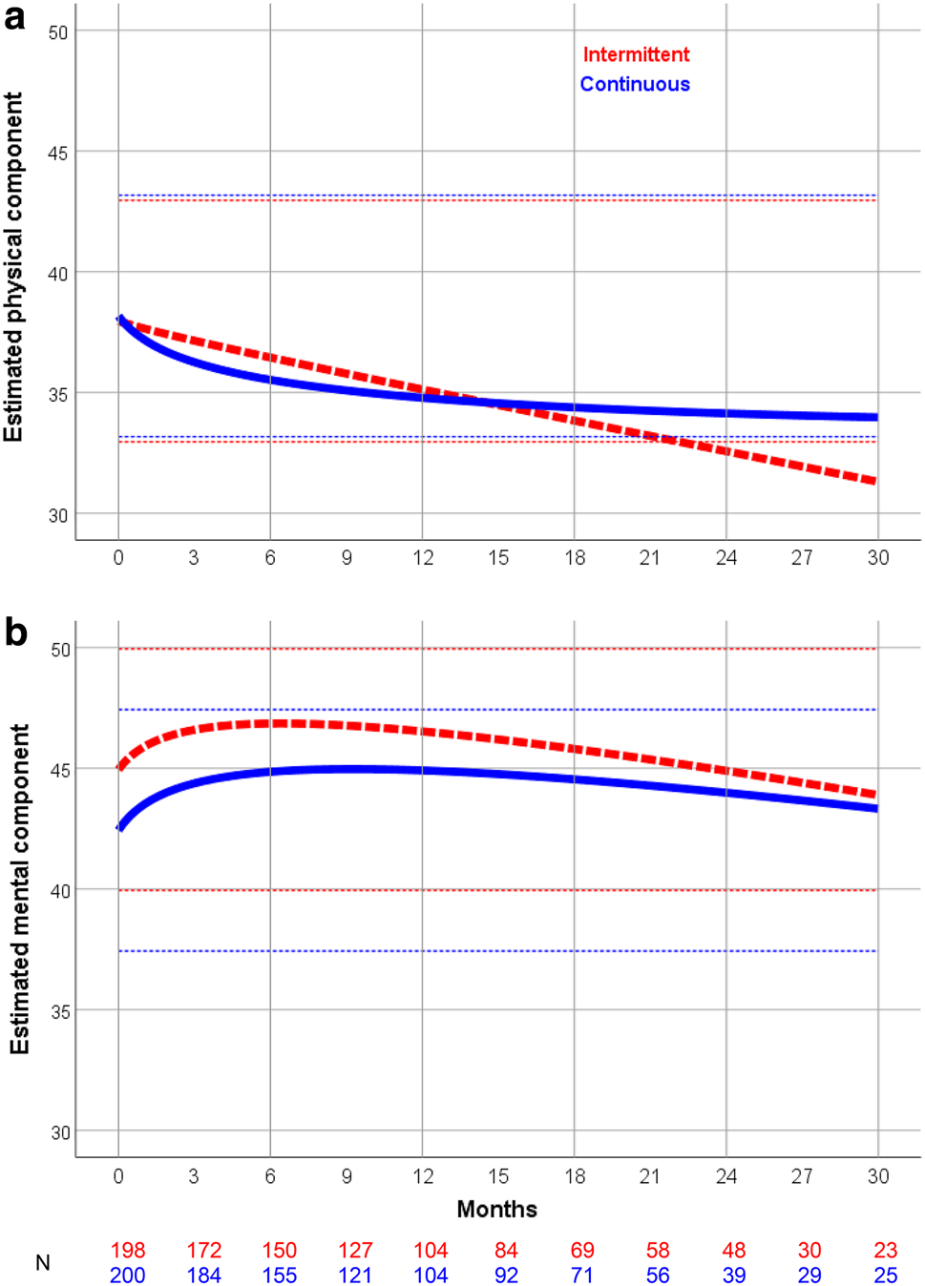
Course of the physical (**a**) and mental (**b**) components of the RAND-36 for both treatment groups. The red dotted lines represent intermittent treatment. The blue lines represent continuous treatment. Bold lines represent the estimates. The dotted light lines represent a difference of 5 points with the baseline score for both intermittent treatment (red) and continuous treatment (blue), which are considered to be clinically meaningful. The T-scale on the *Y*-axis is stretched. The T-scale has a mean on average of 50, and a SD of 10. Higher scores indicate better QoL

In the intermittent arm there was an almost linear decline of physical QoL causing a clinically meaningful difference of 5.40 points at 24 months (*p* < 0.001), while scores in the continuous arm stabilized after a decline of ± 3.4 points at 12 months (Table 2, Fig. 2a). Considering mental QoL (Table 2, Fig. 2b), a lower baseline score was observed in the continuous arm (± 2.5 points, *p* = 0.021) compared to the intermittent arm. Both arms showed an increase in mental health after start of the study treatment (1.58 points; *p* = 0.005 and 2.48 points; *p* < 0.001 at 12 months for intermittent and continuous treatment). Similar to the course of physical QoL, the continuous arm had slightly more stable mental QoL scores than the intermittent arm, although for both arms there were no clinically meaningful changes compared to baseline.

When comparing the arms for both components in changes from baseline, the maximum differences were 2.46 points (*p* = 0.101) for physical and 1.95 points (*p* = 0.182) for mental scores, both measured at 30 months and in favor of continuous treatment.

### Secondary analyses

Age and BMI had no significantly differential effect on the course of both physical and mental QoL from baseline for the two treatment arms. Duration of first-line treatment did not affect physical and mental QoL scores over time. Regarding the social status of the patients, there were no significant differences between patients that lived alone and those living together for both physical and mental QoL. Likewise, the duration and number of (re)hospitalizations and the treatment line had no effect on both the physical and mental QoL scores over time. The 23 patients with a baseline ECOG PS score of 2 had 13.1-point lower baseline scores on the physical component (*p* < 0.001) compared to patients with better performance status. These scores increased slightly until 6.8 months, where after there was a large decrease. No significant differences in physical QoL between the treatment arms were observed within this group compared to patients with better performance status (data not shown). Regarding the mental component score, no significant differences were found at all. Concerning the influence of DFI between initial and metastatic diagnosis, patients within the continuous arm had a 0.04 point lower physical component baseline score for each additional month between the initial and metastatic diagnosis (*p* = 0.007). No further differences were found. For the mental score no relevant significant differences were observed.

## Discussion

In this study we found no significant differences between intermittent and continuous first- and second-line chemotherapy for changes from baseline of both physical and mental QoL in patients with HER2-negative advanced breast cancer. Interestingly, when looking at the physical QoL scores in the treatment arms separately, the total decline was considered a clinically meaningful medium effect in the intermittent arm (decline > 5 points on the component score) and a small, clinically not meaningful (< 5 points) effect in the continuous arm (Table 2, Fig. 2).

Although the differences in course of QoL scores between treatment arms in our current study are not statistically significant nor clinically meaningful, a trend for worse scores on the long term can be observed to the detriment of intermittent treatment (Table 2). However, the number of patients still responding to questionnaires at 30 months is small (*n* = 48), which limits the clinical significance due to selection of patients with more favorable prognosis (Figs. 1, 2). Nevertheless, the observed trend accompanies our previous observation based on efficacy results in a consistent manner, underlining the conclusion that continuous treatment was more effective in prolonging survival and did not compromise QoL.

Mental component scores were generally better than physical component scores for both arms. There was even a slight increase in mental QoL for both treatment arms after the start of study treatment (Table 2, Fig. 2b). Speculatively this might be due to a drop in mental QoL before the study, when the patients received their metastatic breast cancer diagnosis. Psychological coping with this new prospect results in a return to the pre-diagnosis mental state. A similar phenomenon, a temporal drop in mental QoL after a severe diagnosis, has been observed in other studies [18–21].

Our observation that continuous scheduling of chemotherapy for patients with advanced breast cancer did not jeopardize QoL—in contrast to shorter interrupted chemotherapy schedules—has equivalents in literature. Five [22–26] out of six [27] randomized controlled trials on longer versus shorter successive durations of chemotherapy for advanced breast cancer measuring QoL found comparable or better QoL with longer durations of chemotherapy. The study of Coates and colleagues resembles our study best [25]. There, patients were randomized to three cycles of chemotherapy intermittently, versus continuous chemotherapy, both until disease progression occurred. Although the outdated chemotherapy regimen used in that study limits its relevance today, Coates also found worse QoL scores for intermittent compared to continuous treatment [25].

Strengths of this study include the evaluation of QoL alongside efficacy endpoints, something often neglected by investigators within this particular field [28]. Studies that do not include QoL analyses can be considered to have an important shortcoming, as outcomes relevant for the patients are missing. Oncologic studies that do include QoL outcomes mostly use standardized questionnaires (e.g., QLQ-C30, QLQ-BR23 and FACT-B) that also focus on disease-specific symptoms, e.g., for breast cancer patients related to local breast surgery, body image and sexual functioning [28]. In comparison, the RAND-36 used here may be too generic to detect subtle differences between the two treatment groups. On the other hand, one can expect more general effects of intermittent therapy on the QoL than just the local, disease-specific symptoms. Indeed, the idea was that intermittent therapy should have a positive effect on work- and other daily activities, especially in the earlier lines of chemotherapy. We therefore chose to involve a generic questionnaire, which measures QoL from a broad perspective, at the risk of missing the influence of important facets such as neuropathy [29]. Additionally, the RAND-36 is relatively short and has high interval validity and sensitivity to fluctuations in general health. It is widely used, and therefore the results are comparable with many other studies [10].

Although our trial was not powered to proof significant differences in the secondary endpoints (including predefined QoL outcomes), post-hoc power calculations revealed that the differential effects that could have significantly been found, were smaller than the ‘minimal important difference’ of ± 0.5 SD reported in literature on QoL research [16]. For the physical component, a differential effect of 0.301 SD between the treatment arms could have been found applying two measurement points, and an effect of 0.301 SD applying four measurement points. For the mental component, these effects were 0.327 SD and 0.333 SD, respectively. Therefore, our interpretation of the outcome of the study is not hampered by a lack of power.

In conclusion, intermittent chemotherapy showed a trend for worse impact on QoL compared to continuous chemotherapy in advanced breast cancer. In combination with the previously reported reduced efficacy, we therefore do not recommend this strategy in first- and second-line chemotherapy for patients with HER2-negative advanced breast cancer.

## Electronic supplementary material

Below is the link to the electronic supplementary material.

Supplementary material 1 (DOC 52 kb)

## Data Availability

Additional data on the trial protocol can be found at the EU Clinical Trials Register, using number 2010-021519-18 (https://www.clinicaltrialsregister.eu/ctr-search/search?query=2010-021519-18). The datasets generated and analyzed during the current study are not publicly available but are available from the corresponding author on reasonable request.

## References

[1] Claessens AKM, Timman R, Busschbach JJ, Bouma JM, Rademaker-Lakhai JM, Erdkamp FLG, Tjan-Heijnen VCG, Bos MMEM (2019). 159P_PRInfluence on quality of life of chemotherapy scheduling for patients with advanced HER2-negative breast cancer. Annal Oncol.

[2] EBCc (2019). Continuous chemotherapy improves outcomes and quality of life in advanced breast cancer.

[3] Ferlay J, Soerjomataram I, Dikshit R, Eser S, Mathers C, Rebelo M, Parkin DM, Forman D, Bray F (2015). Cancer incidence and mortality worldwide: sources, methods and major patterns in GLOBOCAN 2012. Int J Cancer.

[4] O’Shaughnessy J (2005). Extending survival with chemotherapy in metastatic breast cancer. Oncologist.

[5] Sant M, Chirlaque Lopez MD, Agresti R, Sanchez Perez MJ, Holleczek B, Bielska-Lasota M, Dimitrova N, Innos K, Katalinic A, Langseth H (2015). Survival of women with cancers of breast and genital organs in Europe 1999–2007: results of the EUROCARE-5 study. Eur J Cancer.

[6] Noone A, Howlader N, Krapcho M, Miller D, Brest A, Yu M, Ruhl J, Tatalovich Z, Mariotto A, Lewis D et al: SEER cancer statistics review 1975–2015. In: Based on November 2017 SEER data submission, posted to SEER webiste April 18. Bethesda, MD: National Cancer Insitute; 2018

[7] European School of Oncology PO: Global status of advanced/metastatic breast cancer: 2005-2015 decade report. In: Advanced breast cancer international consensus conference (ABC). March 2016

[8] Claessens AKM, Bos M, Lopez-Yurda M, Bouma JM, Rademaker-Lakhai JM, Honkoop AH, de Graaf H, van Druten E, van Warmerdam LJC, van der Sangen MJC (2018). Intermittent versus continuous first-line treatment for HER2-negative metastatic breast cancer: the Stop and Go study of the Dutch Breast Cancer Research Group (BOOG). Breast Cancer Res Treat.

[9] Hays RD, Sherbourne CD, Mazel RM (1993). The RAND 36-Item Health Survey 1.0. Health Econ.

[10] Zee KIvd, Sanderman R: Het meten van de algemene gezondheidstoestand met de RAND-36: een handleiding. In. Edited by SHARE RI, Groningen UR, vol. Noordelijk Centrum voor Gezondheidsvraagstukken, NCG.—(NCG reeks Meetinstrumenten; 3) Groningen: Research Institute SHARE; 2012

[11] Aaronson NK, Müller M, Cohen PDA, Essink-Bot ML, Fekkes M, Sanderman R, Sprangers MAG (1998). Velde tA, Verrips E: translation, validation, and norming of the Dutch language version of the SF-36 health survey in community and chronic disease populations. J Clin Epidemiol.

[12] Ware JE, Kosinski M, Keller SK: SF-36 physical and mental health summary scales: a user’s manual. In. Edited by Institute TH. Boston, Massachusetts: New England Medical Centre; 1994

[13] Ware J, Snoww K, Ma K, Bg G: SF36 Health Survey: Manual and Interpretation Guide, vol. 30; 1993

[14] Verbeke G (2000). Linear mixed models for longitudinal data.

[15] Singer J, Willett J (2003). Applied longitudinal data analysis—modeling change and event occurrence.

[16] Norman GR, Sloan JA, Wyrwich KW (2003). Interpretation of changes in health-related quality of life: the remarkable universality of half a standard deviation. Med Care.

[17] Cohen J (1992). A power primer. Psychol Bull.

[18] Jones SMW, LaCroix AZ, Li W, Zaslavsky O, Wassertheil-Smoller S, Weitlauf J, Brenes GA, Nassir R, Ockene JK, Caire-Juvera G (2015). Depression and quality of life before and after breast cancer diagnosis in older women from the Women’s Health Initiative. J Cancer Survivor.

[19] Leung J, Pachana NF, McLaughlin D (2014). Social support and health-related quality of life in women with breast cancer: a longitudinal study. Psychooncology.

[20] Tibben A, Timman R, Bannink EC, Duivenvoorden HJ (1997). Three-year follow-up after presymptomatic testing for Huntington’s disease in tested individuals and partners. Health Psychol.

[21] Wang SY, Hsu SH, Gross CP, Sanft T, Davidoff AJ, Ma X, Yu JB (2016). Association between time since cancer diagnosis and health-related quality of life: a population-level analysis. Value Health.

[22] Park YH, Jung KH, Im SA, Sohn JH, Ro J, Ahn JH, Kim SB, Nam BH, Oh DY, Han SW (2015). Quality of life (QoL) in metastatic breast cancer patients with maintenance paclitaxel plus gemcitabine (PG) chemotherapy: results from phase III, multicenter, randomized trial of maintenance chemotherapy versus observation (KCSG-BR07-02). Breast Cancer Res Treat.

[23] Tredan O, Follana P, Moullet I, Cropet C, Trager-Maury S, Dauba J, Lavau-Denes S, Dieras V, Beal-Ardisson D, Gouttebel M (2016). A phase III trial of exemestane plus bevacizumab maintenance therapy in patients with metastatic breast cancer after first-line taxane and bevacizumab: a GINECO group study. Ann Oncol.

[24] Nooij MA, de Haes JCJM, Beex LVAM, Wildiers J, Klijn J, Becquart D, Jassem J, Engelsman E, Duchateau L (2003). Continuing chemotherapy or not after the induction treatment in advanced breast cancer patients. Clinical outcomes and oncologists’ preferences. Eur J Cancer.

[25] Coates A, Gebski V, Bishop JF, Jeal PN, Woods RL, Snyder R, Tattersall MH, Byrne M, Harvey V, Gill G (1987). Improving the quality of life during chemotherapy for advanced breast cancer. A comparison of intermittent and continuous treatment strategies. N Engl J Med.

[26] Gligorov J, Doval D, Bines J, Alba E, Cortes P, Pierga JY, Gupta V, Costa R, Srock S, de Ducla S (2014). Maintenance capecitabine and bevacizumab versus bevacizumab alone after initial first-line bevacizumab and docetaxel for patients with HER2-negative metastatic breast cancer (IMELDA): a randomised, open-label, phase 3 trial. Lancet Oncol.

[27] Becher R, Kloke O, Hayungs J, Hartwich G, Bartels H, Szanto J, Wolf E, Illiger H-J, Halabi S, Rieche K (1996). Epirubicin and ifosfamide in metastatic breast cancer. Semin Oncol.

[28] Ghislain I, Zikos E, Coens C, Quinten C, Balta V, Tryfonidis K, Piccart M, Zardavas D, Nagele E, Bjelic-Radisic V (2016). Health-related quality of life in locally advanced and metastatic breast cancer: methodological and clinical issues in randomised controlled trials. Lancet Oncol.

[29] Chari N (2013). Metastatic breast cancer in Canada: the lived experiene of patients and caregivers presented by the Canadian Breast Cancer Network and Rethink Breast Cancer. Breast.

