# Supplementary material for: The influence on quality of life of intermittent scheduling in first- and second-line chemotherapy of patients with HER2-negative advanced breast cancer
Source: Breast Cancer Res Treat. 2019 Nov 28;179(3):677–85. doi: 10.1007/s10549-019-05495-3 (PMC6997247; doi:10.1007/s10549-019-05495-3)
Supplement: Supplementary file 1 — Supplementary material 1 (DOC 52 kb) [file 10549_2019_5495_MOESM1_ESM.doc]

# Electronic Supplementary Material

**The influence on quality of life of intermittent scheduling in first- and second-line chemotherapy of patients with HER2-negative advanced breast cancer**

Anouk KM Claessens ^1,5^, Reinier Timman ^2^, Jan J Busschbach ^2^, Jeanette M Bouma ^3^, Jeany M Rademaker-Lakhai ^4^, Frans LG Erdkamp ^1^, Vivianne CG Tjan-Heijnen ^5^ and Monique EMM Bos ^6^ on behalf of the Dutch Breast Cancer Research Group (BOOG)

1. Zuyderland Medical Centre, Department of Medical Oncology, Dr. H. van der Hoffplein 1, 6162 BG Geleen, The Netherlands, [a.claessens@zuyderland.nl](mailto:a.claessens@zuyderland.nl) / [f.erdkamp@zuyderland.nl](mailto:f.erdkamp@zuyderland.nl)
2. Erasmus Medical Centre, Department of Psychiatry, section of Medical Psychology & Psychotherapy, Doctor Molewaterplein 40, 3015 GD Rotterdam, the Netherlands, [r.timman@erasmusmc.nl](mailto:r.timman@erasmusmc.nl) / [j.vanbusschbach@erasmusmc.nl](mailto:j.vanbusschbach@erasmusmc.nl)
3. Comprehensive Cancer Centre the Netherlands, Department of trial registration, Vasteland 78, 3011 BN Rotterdam, the Netherlands, [j.bouma@iknl.nl](mailto:j.bouma@iknl.nl)
4. Dutch Breast Cancer Research Group, BOOG study Center, IJsbaanpad 9, 1076 CV Amsterdam, the Netherlands, [j.rademaker@boogstudycenter.nl](mailto:j.rademaker@boogstudycenter.nl)
5. Maastricht University Medical Centre, Department of Medical Oncology, GROW – School for Oncology and Developmental Biology, Maastricht, the Netherlands, P. Debyelaan 25, 6229 HX Maastricht, the Netherlands, [vcg.tjan.heijnen@mumc.nl](mailto:vcg.tjan.heijnen@mumc.nl)
6. Erasmus Medical Centre, Department of Medical Oncology, Doctor Molewaterplein 40, 3015 GD Rotterdam, the Netherlands, [m.bos@erasmusmcl.nl](mailto:m.bos@erasmusmcl.nl)

**Corresponding author**: Monique E.M.M. Bos, M.D. Ph.D., Medical Oncologist, Erasmus Medical Centre, Department of Medical Oncology, Doctor Molewaterplein 40, 3015 GD Rotterdam, the Netherlands, [m.bos@erasmusmc.nl](mailto:m.bos@erasmusmc.nl)


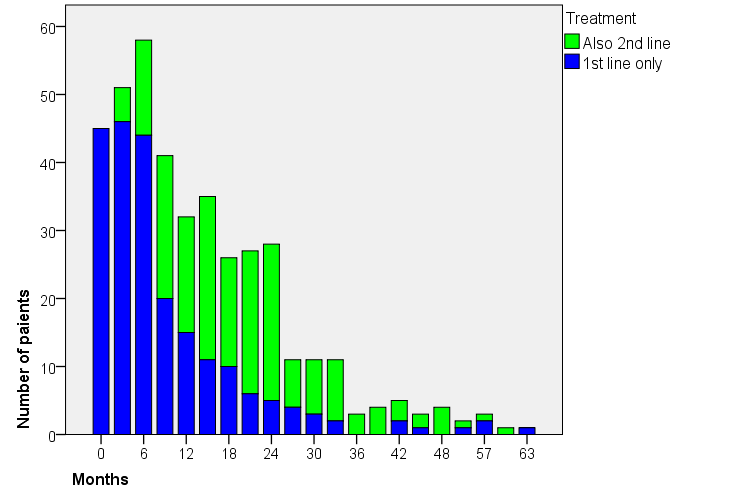


## Supplementary Fig 1 Maximum follow-up time per patient in first- and second-line study treatment

**NOTE**: The maximum follow-up time for QoL is given on the X-axis in months. The number of patients is given on the Y-axis. The blue bars represent patients who only received first-line study treatment. The green bars represent patients who also received second-line study treatment. For example, bar 0 counts 42 patients who only have baseline QoL scores. Bar 6 counts 57 patients with a maximum follow-up of 6 months, of whom 43 patients only received first-line study treatment and 14 patients also received second-line study treatment.

## Supplementary Table 1. Models of primary analyses, with the continuous treatment arm as reference group.

|  |  | **Physical component score** | | | | **Mental component score** | | | |  |
| --- | --- | --- | --- | --- | --- | --- | --- | --- | --- | --- |
|  | Effect | estimate | 95% CI | | p-value | estimate | 95% CI | | p-value |  |
|  | Intercept | 38.17 | 36.73 | 39.62 | <0.001 | 42.43 | 40.93 | 43.94 | <0.001 |  |
|  | Linear time | 0.02 | -0.10 | 0.14 | 0.690 | -0.17 | -0.30 | -0.05 | 0.008 |  |
|  | Log time | -1.43 | -2.25 | -0.62 | 0.001 | 1.78 | 0.86 | 2.70 | <0.001 |  |
|  | Intermittent | -0.21 | -2.26 | 1.84 | 0.842 | 2.51 | 0.37 | 4.65 | 0.021 |  |
|  | Intermittent * linear time | -0.23 | -0.39 | -0.07 | 0.006 | -0.05 | -0.23 | 0.12 | 0.545 |  |
|  | Intermittent * log time | 1.29 | 0.15 | 2.43 | 0.026 | -0.10 | -1.37 | 1.18 | 0.881 |  |
|  |  | | | | | | | | |  |
